# Pattern and predictors of maternal care-seeking practices for severe neonatal jaundice in Nigeria: a multi-centre survey

**DOI:** 10.1186/1472-6963-14-192

**Published:** 2014-04-28

**Authors:** Chinyere V Ezeaka, Rosemary O Ugwu, Mariya Mukhtar-Yola, Ekanem N Ekure, Bolajoko O Olusanya

**Affiliations:** 1 Department of Paediatrics, Lagos University Teaching Hospital, Surulere, Lagos, Nigeria; 2 Department of Paediatrics and Child Health, University of Port Harcourt Teaching Hospital, Port Harcourt, Nigeria; 3 Department of Paediatrics, National Hospital, Abuja, Nigeria; 4 Centre for Healthy Start Initiative, Dolphin Estate, Ikoyi, Lagos, Nigeria

**Keywords:** Neonatal jaundice, Newborn care, Health-seeking behaviour, Health promotion, Haemolytic agents, Self-medication, Developing countries

## Abstract

**Background:**

Nigeria is frequently associated with disproportionately high rates of severe neonatal jaundice (NNJ) underpinned by widespread Glucose-6-phosphate dehydrogenase (G6PD) deficiency. Timely and appropriate treatment of NNJ is crucial for preventing the associated morbidity and neuro-developmental sequelae. Since mothers are likely to be the first mostly to observe the onset of severe illness in their newborns, we set out to identify the pattern and predictors of maternal care-seeking practices for NNJ in three culturally-distinct settings in Nigeria.

**Methods:**

A multi-centre study was conducted among women attending antenatal clinics in Abuja, Lagos and Port Harcourt from October 2011 to April 2012 using a pretested questionnaire. Predictors of awareness of NNJ, accurate recognition of NNJ, use of potentially harmful therapies and preference for future hospital treatment were determined with multivariate logistic regressions.

**Results:**

Of the 488 participants drawn from the three locations, 431 (88.3%) reported awareness of NNJ, predominantly (57.8%) attributable to professional health workers. A total of 309 (63.3%) mothers with prior knowledge of NNJ claimed they could recognise NNJ, but 270 (87.4%) from this group accurately identified the features of NNJ. Multiparous mothers (Adjusted odds ratio, AOR:4.05; 95% CI:1.75-9.36), those with tertiary education (AOR:1.91; CI:1.01-3.61), and those residing in Lagos (AOR:2.96; CI:1.10-7.97) were more likely to have had prior knowledge of NNJ. Similarly, multiparous mothers (AOR:2.38; CI:1.27-4.46) and those with tertiary education (AOR:1.92; CI:1.21-3.05) were more likely to recognise an infant with jaundice accurately. Mothers educated by health workers were 40% less likely to resort to potentially harmful treatment for NNJ (AOR:0.60; CI:0.39-0.92) but more likely to seek hospital treatment in future for an infant suspected with jaundice (AOR:1.88; CI:1.20-2.95).

**Conclusions:**

Women with tertiary education and multiparous mothers who attend routine antenatal clinics are more likely than less educated women, to be associated with appropriate care-seeking practices for infants with NNJ regardless of the socio-cultural setting. Systematic efforts by professional health workers are warranted, as part of routine antenatal care, to engage other groups of mothers especially those likely to indulge in self-use of potentially harmful therapies.

## Background

Severe neonatal jaundice or hyperbilirubinaemia (NNJ) resulting from excessive unconjugated bilirubin levels is the commonest or one of the five leading conditions for hospital (re)admissions in the first week of life in many low-income countries [1]. If poorly managed, severe NNJ may result in deaths [2-5], or various lifelong neuro-developmental impairments such as intellectual deficits, cerebral palsy, sensorineural hearing loss, epilepsy and behavioural problems among survivors [5-7].

In the majority of newborns with uncomplicated vaginal delivery, NNJ commonly manifests 48 hours after birth and hospital discharge. Mothers are thus, likely to be the first to observe the onset of NNJ in affected infants. Poor or lack of understanding of this condition is likely to result in risky delays, mismanagement and complications with adverse psycho-social consequences for the affected mothers [8]. With appropriate education, mothers can reliably recognise the familiar discolouration of the sclera and mucous membranes and seek timely treatment [9]. Informed and appropriate care-seeking practices by mothers are, therefore, recognised worldwide as an integral component of the effective management of NNJ.

The burden of NNJ underpinned by widespread Glucose-6-phosphate dehydrogenase (G6PD) deficiency has been extensively reported for several years in Nigeria [4,5,10-15]. About 5.5% of all newborns are estimated to have clinically significant NNJ requiring phototherapy and/or exchange blood transfusion (EBT) in the country, probably one of the highest rates globally [16]. Most tertiary institutions are still overwhelmed with exceptionally high rates of EBT daily [17]. The role of local cultural practices in the (mis)management of NNJ has also been widely reported [10,18]. However, only limited studies have examined factors associated with appropriate care-seeking disposition for NNJ among expectant mothers across distinct socio-cultural settings in Nigeria [19-21]. This multi-centre study, therefore, set out to assess the level of awareness and understanding of NNJ among pregnant mothers as well as identify predictors of appropriate care-seeking practices to facilitate early detection and intervention for infants at risk of severe NNJ in Nigeria.

## Methods

This cross-sectional study was carried out at the three tertiary public hospitals located in Abuja (Northern Nigeria), Lagos (Southwest, Nigeria) and Port Harcourt (Southeast, Nigeria) between October 2011 and April 2012. The sites are described by their approximate geographical locations rather than geopolitical zones. Abuja is the administrative capital and the seat of the federal government while Lagos is the most commercially active city in Nigeria. Port Harcourt is the most prominent city in the oil producing Niger-Delta region of the country. The survey instrument was pretested among mothers in one of the tertiary centres between January and April 2009, evaluated independently by a team of experienced paediatricians and appropriately revised into a concise one-page document for ease of administration before the commencement of the multi-centre study (Additional file 1). Three of the authors personally supervised the administration of the questionnaire at their respective centres having also contributed to the final construct of the questionnaire for comprehension and content validity. Medical and other terminologies were clearly explained by the interviewer to the participants especially those who were not literate. The required minimum sample size after allowing for 20% attrition at 95% confidence interval was 380. This was computed in Epi-Info 7.0 (CDC, Atlanta, USA) based on the total population of women in the three locations where the study was conducted as per the 2006 national census data and the weighted mean prevalence of 71.1% derived from relevant local research [19-21].

Ethical approval was obtained from the Lagos University Teaching Hospital, Lagos, Nigeria (the lead/coordinating institution) for the study design which did not entail any prophylactic, diagnostic or therapeutic interventions. In line with the Helsinki Declaration [22], only mothers from whom informed consent was obtained were enrolled for the study. The questionnaire was administered to consecutive mothers who attended the antenatal clinic at each centre. The term “mothers” was used broadly in this study for first-time mothers and those already with children. The first part of the questionnaire included socio-demographic data of respondents such as maternal age, marital status, parity, ethnicity, religion, self and spouse’s education, occupation, residential type (an indicator of neighbourhood influence) and home ownership (an indicator of economic status). The next part aimed to assess the knowledge of the mothers and the source of information, accurate recognition of NNJ, knowledge of the potential dangers or complications of severe NNJ, treatment modalities or preferences. The potential dangers considered included death or neuro-developmental delay or disability such as motor deficit, delayed milestones, mental retardation, speech defects or deafness. The third part sought to establish the respondent’s experience with an infant with jaundice and the actions taken as well as possible future course of action if confronted with a jaundiced infant.

An overview of the socio-demographic characteristics of the respondents across the three centres was examined with descriptive statistics. Logistic regression models were used to predict factors significantly associated with four main outcomes. Firstly, we examined factors predictive of prior knowledge of NNJ among all respondents (n = 488). Secondly, we constructed three separate models to identify predictors of accurate recognition of NNJ, self-treatment with potentially harmful substances and future hospital visit for suspected NNJ among women with prior knowledge of NNJ (n = 431). Maternal recognition of NNJ was considered as accurate if yellowish discolouration of the eyes and/or face and body skin along the cephalocaudal progression were mentioned. Two-tailed p-values equal to or less than 0.05 were considered as significant. However, selection of variables for the models was not restricted to those with significant outcomes from bivariate analysis but included those closely linked with maternal health seeking behaviour in previous local studies [19-21,23]. Strength of association in each model was estimated by adjusted odds ratios (AOR) and the corresponding 95% confidence intervals (CI). Model calibration or goodness-of-fit was verified with the Hosmer-Lemeshow test. Except for sample size calculation, IBM SPSS Statistics for Windows, Version 20.0 (Armonk, NY: IBM Corp) was used for all statistical analyses.

## Results

### Characteristics of respondents

**Figure 1:**
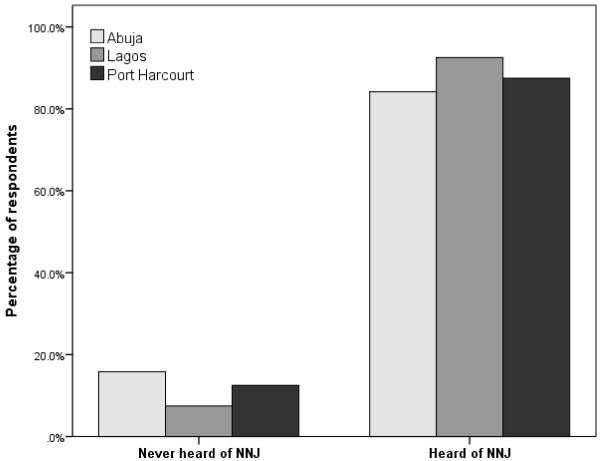
Proportion of respondents (n = 488) with or without prior knowledge of neonatal jaundice across the survey centres.

A total of 488 women completed the questionnaire from the three centres out of which 57 (11.7%) women said they had no knowledge of NNJ. Of the 431 women who have some knowledge of NNJ, 85 (19.7%) were in Abuja, 136 (31.6%) in Lagos and 210 (48.7%) in Port Harcourt. The proportion of women who have not or have heard of NNJ among the respondents in each location is shown in Figure 1. The proportion of women who had no prior knowledge of NNJ among respondents was highest in Abuja (15.8%) and lowest in Lagos (7.5%). The socio-demographic profile of the women with prior knowledge of NNJ across the three locations is presented in Table 1. Majority of respondents were 20-35 years of age, married, had 2 or more children, belonged to one of the three major tribes (i.e. Hausa, Ibo and Yoruba), professed Christianity, had tertiary education and lived in self-contained but rented residences.

**Table 1:** Characteristics of pregnant mothers enrolled for study at three centres (n = 431)

**Factors**	**Abuja**	**Lagos**	**Port Harcourt**	**Total**
	**n = 85**	**n = 136**	**n = 210**	**n (%)**
Maternal age (Years)				
< 20	0	2	0	2 (0.5)
20 – 35	85	100	199	384 (89.1)
>35	0	14	11	25 (5.8)
Unknown	0	20	0	20 (4.6)
Marital status				
Single	5	3	10	18 (4.1)
Married	77	133	198	408 (94.7)
Separated/Widow	3	0	2	5 (1.2)
Parity				
0	3	58	24	85 (19.7)
1	28	39	56	123 (28.5)
2 or more	54	39	130	223 (51.7)
Ethnicity				
Hausa	20	17	19	56 (13.0)
Ibo	25	42	58	125 (29.0)
Yoruba	15	57	12	84 (19.5)
Others	25	20	121	166 (38.5)
Religion				
Christianity	76	111	204	391 (90.7)
Islam	9	25	6	40 (9.3)
Education				
Primary	5	4	9	18 (4.2)
Secondary	24	21	62	107 (24.9)
Technical	10	9	24	43 (10.0)
Tertiary	46	102	115	263 (61.0)
Education of spouse				
Primary	1	3	4	8 (1.9)
Secondary	13	32	44	89 (20.6)
Technical	10	5	17	32 (7.4)
Tertiary	61	96	145	302 (70.1)
Occupation				
None	9	12	46	67 (15.5)
Student	1	12	20	33 (7.7)
Self-employed	37	41	74	152 (35.3)
Formal Job	32	56	65	153 (35.5)
Unknown	6	15	5	26 (6.0)
Residential type				
Self-contained	60	110	168	338 (78.4)
Shared	15	26	40	81 (18.8)
Unknown	10	0	2	12 (27.8)
Home ownership status				
Owned	26	20	62	108 (25.0)
Rented	49	116	146	311 (72.2)
Unknown	10	0	2	12 (27.8)

### Characteristics of respondents with knowledge of newborn jaundice

Of all the 431 mothers with prior knowledge of NNJ, 249 (57.8%) ascribed this information to health workers (doctors or nurses) as shown in Table 2. While 309 (71.7%) mothers reported ability to recognise NNJ, only 87.4% (270/309) of these mothers demonstrated accurate recognition of NNJ representing 62.6% of those who claimed prior knowledge of NNJ. A total of 240 mothers, representing 77.7% of mothers who reported their ability to recognise NNJ or 55.7% of those with reported prior knowledge of NNJ recognised the effect of NNJ as poor feeding, irritability, abnormal cry, abnormal body stretching or abnormal eye movement. Of all mothers with prior NNJ awareness, 314 (72.8%) mothers cited the dangers of NNJ as either death, disability or both. Few (35 or 8.1%) mothers attributed NNJ to infections, prematurity or ABO/Rh incompatibilities. Although the vast majority (91.9%) of the mothers planned to deliver the index pregnancies in hospitals, more than half (55.8%) indicated the possible use of potentially harmful menthol substances either as treatment for NNJ or some other purposes.

**Table 2:** Knowledge of NNJ among mothers attending routine prenatal clinics in Nigeria (n = 431)

**Responses**	**Abuja**	**Lagos**	**Port Harcourt**	**Total**
	**n = 85 (%)**	**n = 136 (%)**	**n = 210 (%)**	**n (%)**
Heard about jaundice from				
Friend/Neighbour	15 (17.6)	37 (27.2)	46 (21.9)	98 (22.7)
Health worker	57 (67.1)	61 (44.9)	131 (62.4)	249 (57.8)
Relations	5 (5.9)	16 (11.8)	12 (5.7)	33 (7.7)
Media	1 (1.2)	5 (3.7)	7 (3.3)	13 (3.0)
Other	7 (8.2)	17 (12.5)	14 (6.7)	38 (8.8)
Can recognise baby with jaundice				
No	17 (20.0)	47 (34.6)	58 (27.6)	122 (28.3)
Yes	68 (80.0)	89 (65.4)	152 (72.4)	309 (71.7)
Where to look for in the baby				
Eye, body or skin	68 (80.0)	89 (65.4)	152 (72.4)	309 (71.7)
Not stated	17 (20.0)	47 (34.6)	58 (27.6)	122 (28.3)
What would be seen in baby				
Yellowish discolouration	53 (62.4)	75 (55.1)	142 (67.6)	270 (62.6)
Other	13 (15.3)	11 (8.1)	10 (4.8)	34 (7.9)
Not stated	19 (22.4)	50 (36.8)	58 (27.6)	127 (29.5)
Possible effects of severe jaundice in a baby				
Poor feeding	8 (9.4)	28 (20.6)	58 (27.6)	94 (21.8)
Irritable	4 (4.7)	12 (8.8)	11 (5.2)	27 (6.3)
Abnormal cry	15 (17.6)	12 (8.8)	22 (10.5)	49 (11.4)
Abnormal body stretching	6 (7.1)	12 (8.8)	12 (5.7)	30 (7.0)
Abnormal eye movement	9 (10.6)	15 (11.0)	16 (7.6)	40 (9.3)
Other	7 (8.2)	2 (1.5)	6 (2.9)	15 (3.5)
Not stated	36 (42.4)	55 (40.4)	85 (40.5)	176 (40.7)
Possible dangers of jaundice				
Death	22 (25.9)	71 (52.2)	65 (31.0)	158 (36.6)
Disability	30 (35.3)	21 (15.4)	49 (23.3)	100 (23.2)
Death/Disability	7 (8.2)	13 (9.6)	36 (17.1)	56 (13.0)
Other	3 (3.5)	3 (2.2)	9 (4.3)	15 (3.5)
Not stated	23 (27.1)	28 (20.6)	51 (24.3)	102 (23.7)
Possible causes of jaundice				
Infections	15 (17.6)	2 (1.5)	9 (4.3)	26 (6.0)
Malaria/Fever	23 (27.1)	31 (22.8)	74 (35.2)	128 (29.7)
Prematurity	0 (0.0)	0 (0.0)	6 (2.9)	6 (1.4)
ABO/Rhesus incompatibility	0 (0.0)	1 (0.7)	2 (1.0)	3 (0.7)
Others	18 (21.2)	11 (8.8)	27 (12.9)	56 (13.0)
Not stated	29 (34.1)	91 (66.9)	92 (43.8)	212 (49.2)
Planned place of delivery				
Same hospital	51 (60.0)	113 (83.1)	158 (75.2)	322 (74.7)
Other public hospital	20 (23.5)	20 (14.7)	27 (12.9)	67 (15.5)
Private hospital	14 (16.5)	3 (2.2)	21 (10.0)	38 (8.8)
Outside hospital	0 (0.0)	0 (0.0)	4 (1.9)	4 (0.9)
Possible use of haemolytic substances				
Dusting powder	23 (27.1)	47 (34.6)	62 (29.5)	132 (30.6)
Robb mentholated cream	17 (20.0)	9 (6.6)	13 (6.2)	39 (9.0)
Eucalyptus oil	10 (11.8)	28 (20.6)	15 (7.1)	53 (12.3)
Camphor on clothes	0 (0.0)	5 (3.7)	12 (5.7)	17 (3.9)
Not stated	35 (41.2)	47 (34.6)	108 (51.4)	190 (44.1)

### Care-seeking practices of mothers with knowledge of newborn jaundice

Almost 30% of the mothers (n = 128) claimed to have had an infant with jaundice and health workers were the first to detect this condition in about 44.5% (n = 57) of the affected infants (Table 3). Hospital admission, reported in 70 (16.2%) mothers, was the most common intervention followed by exposure to sunlight by 38 (8.8%) mothers. Although majority (89.1%) of the mothers reported full recovery in the affected infants, 9 (7.0%) mothers reported neonatal deaths and 3 (2.3%) mothers reported recovery with some sequelae. The majority (70.3% or 303/431) of mothers indicated that they would seek advice or hospital care if confronted again with a jaundiced infant. From this group, 96 (31.7%) had an infant with jaundice. Up to 25% (32/128) of mothers with prior experience of an infant with jaundice may not seek hospital care, and about 18% (51/128) indicated sunlight exposure as possible treatment for NNJ. The majority (62.5% or 20/32) of mothers with prior experience of a jaundiced infant attributed NNJ to malaria/fever, and 34.4% (11/32) did not ascribe NNJ to any specific causes. Three quarters (24/32) of this group of mothers cited death and/or disability as possible outcomes of NNJ.

**Table 3:** Experience and planned action by mothers on infants with jaundice (n = 431)

**Responses**	**Abuja**	**Lagos**	**Port Harcourt**	**Total**
	**n = 85 (%)**	**n = 136 (%)**	**n = 210 (%)**	**n (%)**
Ever had baby with jaundice				
No	56 (65.9)	107 (78.7)	140 (66.7)	303 (70.3)
Yes	29 (34.1)	29 (21.3)	70 (33.3)	128 (29.7)
Who first detected the baby with jaundice				
Self	12 (14.1)	6 (4.4)	22 (10.5)	40 (9.3)
Relation/Neighbour	3 (3.5)	9 (6.6)	18 (8.6)	30 (7.0)
Doctor/Nurse	14 (16.5)	13 (9.6)	30 (14.3)	57 (13.2)
Not stated	0 (0.0)	1 (0.7)	0 (0.0)	1 (0.2)
Not applicable	56 (65.9)	107 (78.7)	140 (66.7)	303 (70.3)
Action taken/treatment				
Hospital admission	23 (27.1)	17 (12.5)	15 (14.3)	70 (16.2)
Sunlight exposure	5 (5.9)	5 (3.7)	28 (13.3)	38 (8.8)
Antibiotics	0 (0.0)	0 (0.0)	3 (0.7)	3 (0.7)
Breast feeding	0 (0.0)	1 (0.7)	1 (0.5)	2 (0.5)
Pawpaw	1 (1.2)	0 (0.0)	0 (0.0)	1 (0.2)
Not stated	0 (0.0)	6 (4.4)	8 (3.8)	14 (3.2)
Not applicable	56 (65.9)	107 (78.7)	140 (66.7)	303 (70.3)
Final outcome				
Baby died	1 (1.2)	1 (0.7)	7 (3.3)	9 (2.1)
Baby recovered fully	28 (32.9)	23 (16.9)	63 (30.0)	114 (26.5)
Baby survived with problems	0 (0.0)	3 (2.2)	0 (0.0)	3 (0.7)
Not stated	0 (0.0)	2 (1.5)	0 (0.0)	2 (0.5)
Not applicable	56 (65.9)	107 (78.7)	140 (66.7)	303 (70.3)
Possible future action				
Go to hospital	67 (78.8)	91 (66.9)	145 (69.0)	303 (70.3)
Sunlight exposure	11 (12.9)	1 (0.7)	39 (18.6)	51 (11.8)
Antibiotics	2 (2.4)	0 (0.0)	1 (0.5)	3 (0.7)
Exclusive breastfeeding	0 (0.0)	1 (0.7)	1 (0.5)	2 (0.5)
Not stated	5 (5.9)	43 (31.6)	24 (11.4)	72 (16.7)

### Predictors of prior knowledge of NNJ and care-seeking practices

The first regression model (Table 4) showed that mothers who were significantly more likely to have had prior knowledge of NNJ among all the respondents were those with 2 or more children (AOR:4.05; CI:1.75-9.36), those with tertiary education (AOR:1.91; CI:1.01-3.61), and those residing in Lagos (AOR:2.96; CI:1.10-7.97). The second model suggests that multi-parous mothers (AOR:2.38; CI:1.27-4.46) and those with tertiary education (AOR:1.92; CI:1.21-3.05) were also significantly more likely to recognise an infant with jaundice accurately. In the third model, mothers whose source of information about NNJ was a health worker compared to friends, neighbours or relations that are non-health workers were 40% less likely to resort to the use of potentially harmful substances or therapies for an infant with jaundice (OR:0.60; CI:0.39-0.92). The final model also showed that this group of mothers was significantly more likely in the future to seek hospital treatment for an infant suspected with jaundice (OR:1.88; CI:1.20-2.95). Ethnicity, religion or residential type was not predictive of maternal knowledge of or experience with NNJ. Accurate recognition of NNJ, self-treatment and planned future hospital visit for NNJ were independent of the residential location of the mothers. No factor was also found to be predictive of mothers with prior experience of a jaundiced infant that may not seek hospital care for a future NNJ episode. Maternal age was not considered for inclusion into any of the models as almost 90% of the respondents were within a single age bracket. All models were satisfactorily calibrated as demonstrated by Hosmer-Lemeshow test results.

**Table 4:** Logistic regression models predicting prior knowledge of NNJ, accurate recognition of NNJ, self-treatment and planned future hospital visit for NNJ treatment

**Factors**	**Model 1**	**Model 2**	**Model 3**	**Model 4**
	**AOR (95% Cl)**	**AOR (95% Cl)**	**AOR (95% Cl)**	**AOR (95% Cl)**
Parity				
0	Reference	Reference	Reference	Reference
1	1.76 (0.78-3.96)	1.48 (0.80-2.76)	1.58 (0.85-2.96)	1.86 (0.98-3.55)
2 or more	4.05 (1.75-9.36)***	2.38 (1.27-4.46)**	1.64 (0.89-3.03)	1.63 (0.87-3.04)
Ethnicity				
Hausa	Reference	Reference	Reference	Reference
Ibo	1.11 (0.42-2.97)	0.94 (0.42-2.10)	0.91(0.45-1.83)	1.07 (0.50-2.29)
Yoruba	1.44 (0.49-4.25)	0.55 (0.24-1.24)	1.47 (0.68-3.16)	1.13 (0.50-2.58)
Others	1.49 (0.56-3.98)	0.75 (0.34-1.63)	0.81 (0.41-1.62)	0.90 (0.43-1.91)
Religion				
Christianity	Reference	Reference	Reference	Reference
Islam	0.69 (0.23-2.07)	1.68 (0.72-3.90)	1.08 (0.49-2.35)	2.04 (0.83-5.01)
Education				
Non-tertiary	Reference	Reference	Reference	Reference
Tertiary	1.91 (1.01-3.61)*	1.92 (1.21-3.05)**	0.78 (0.51-1.21)	1.20 (0.74-1.92)
Residential type				
Self-contained	Reference	Not Applicable	Reference	Reference
Shared	0.71 (0.35-1.41)		1.29 (0.76-2.19)	0.70 (0.41-1.22)
Location				
Abuja	Reference	Reference	Reference	Reference
Lagos	2.96 (1.10-7.97)*	0.60 (0.30-1.23)	1.33 (0.70-2.54)	0.67 (0.33-1.40)
Port Harcourt	1.15 (0.53-2.48)	0.70 (0.37-1.34)	0.74 (0.43-1.30)	0.70 (0.36-1.35)
Source of information				
Non health worker	Not applicable	Reference	Reference	Reference
Health worker		1.09 (0.69-1.73)	0.60 (0.39-0.92)*	1.88 (1.20-2.95)**
Hosmer-Lemeshow Test	p = 0.235	p = 0.395	p = 0.769	p = 0.552

*p < 0.05; **p < 0.01; ***p < 001; AOR = adjusted odds ratio; CI = confidence interval.

Model 1: Factors predicting women who are most likely to have previously heard about neonatal jaundice.

Model 2: Factors predicting ability to recognise the possible occurrence of NNJ.

Model 3: Factors predicting self-treatment with potentially harmful substances.

Model 4: Factors predicting women who are likely to seek or advise hospital care for an infant with NNJ.

## Discussion

Our study builds on the substantial body of evidence in the literature on the seemingly intractable but avoidable burden of severe NNJ in Nigeria by exploring a critical pathway to appropriate care-seeking behaviour among pregnant mothers [4,5,10-21]. This is also against the backdrop of recent findings from a national survey of paediatricians in the country which highlighted the apparent omission of NNJ in the current global child health priorities for newborn care for Nigeria and comparable developing countries [24].

This study suggests that there is a high level of awareness of NNJ among women attending antenatal clinics in tertiary hospitals with or without a prior personal experience of a jaundiced infant. This is consistent with findings in similar studies in Nigeria and other developing countries in which awareness of NNJ among women ranges from 86-100% prior to delivery [19,20,25], shortly after delivery [26], or while presenting in hospitals for newborn care [21,27]. About 37% of women who indicated an awareness of NNJ in our study were unlikely to accurately recognise the onset of the condition. One study in Nigeria reported a rate as high as 43% in this group of women [20], while others have reported rates as low as 23% [19,27]. Multiparous mothers were four-times more likely to have a knowledge of NNJ than first-time mothers while women with tertiary education were almost twice more likely to be aware than women with less than tertiary education. The awareness among multiparous and highly educated mothers was also associated with accurate recognition of NNJ in the affected infants which is corroborated by findings in comparable studies in Nigeria and Turkey [19,21,28]. Mothers residing in Lagos appear to have the greatest advantage as they were almost three-times more likely to have heard about NNJ than say mothers residing in Abuja. This may be attributed to the fact that the city only ceded its status as the federal capital since independence in 1960 to Abuja in 1991 and still remains the financial/commercial nerve-centre for the country. However, these mothers were not significantly more likely to recognise NNJ than those outside Lagos suggesting that geographical location had little or no impact on mothers’ accurate recognition of a jaundiced infant. While a high percentage of mothers also recognised the physical symptoms of NNJ, any suggestion of high NNJ awareness among any groups of women must not be misconstrued as ability to recognise the onset of the condition or assurance of appropriate care-seeking decision. A nation-wide campaign to eliminate the differences in the level of awareness across the country is warranted. Perhaps, the greatest challenge is in disseminating this education to the vast majority of women in Nigeria who presently do not deliver in hospitals [16].

It is insightful that many mothers still indicated the use of traditional and potentially harmful therapies regardless of the planned delivery in a health facility for their current pregnancy. This would suggest that the dangers of such practice are yet to be well-appreciated across the country. Some mothers may, in fact, nurse the belief that these traditional therapies are complementary to hospital treatment as “first-aid”. Our study suggests that the quality and source of education on NNJ may, in fact, explain this tendency or practice in the affected mothers. Mothers who derived their knowledge of NNJ from health workers were significantly less inclined to self-treatment and more likely to seek hospital treatment for their jaundiced infants. Thus the observed high level of awareness reported in this and other studies must also not distract from the underlying risks associated with sources of NNJ education that are not health workers. This finding is reassuring as some professional health workers have been blamed for encouraging or perhaps not discouraging direct sunlight exposure as possible treatment for NNJ which is still common in many developing countries [25,27,29,30]. One study in Nigeria reported that 50.7% of mothers who claimed awareness of NNJ indicated exposure to sunlight as their preferred treatment option [27]. Another from Malaysia showed that 83.1% of the mothers from different races practised the sunning of their jaundiced infants [25]. However, there is emerging evidence on the possible utilization of specially “filtered” sunlight phototherapy using tinted films for the safe and effective treatment of NNJ especially in resource-limited communities without (steady) electricity supply to support conventional electric-powered phototherapy units [31]. This holds promise as an improvement on the pioneering effort of an indigenous neonatologist who introduced a “sunshine phototherapy cot” for treatment of mild NNJ in primary health care centres [27,32].

Our study, like prior reports, demonstrates the need for focused educational campaign among the most vulnerable groups of mothers (particularly first-time mothers and those without tertiary education) prior to delivery to improve their knowledge of the risk factors for severe NNJ, the accurate detection of its onset, avoidance of harmful therapies, the benefits of early and appropriate intervention, as well as the potential consequences for case fatality or life-long detrimental effects on the survivors and their families. In fact, even where requisite services are available, the life-time rehabilitation costs for the associated developmental impairments like cerebral palsy, deafness, autism and epilepsy are far beyond the reach of most families in these settings. This consideration itself should provide the impetus for needed preventive action by policy makers at all levels of health care delivery. Efforts to promote proper care-seeking behaviour by mothers must nonetheless be backed and sustained by the provision of effective treatment for those who present in hospitals especially after early (<48hours) hospital discharge which is quite common. For example, there are several reports on the poor quality of treatment offered even in tertiary hospitals as a result of ineffective phototherapy, avoidable exchange blood transfusions and lack of reliable devices for real-time bilirubin monitoring [17,33,34]. This situation sometimes weakens the resolve and enthusiasm of some health workers to promote hospital treatment aggressively, thereby inadvertently fostering recourse to “alternative” traditional therapies including indiscriminate (mis)use of antibiotics such as Ampiclox® and gentamycin. Our study also suggests that about one quarter of mothers with prior experience with a jaundiced infant may not seek hospital care but resort to self-treatment of perceived malaria/fever as the underlying cause of NNJ. We were unable to establish any specific socio-demographic predictors for this group of mothers.

Besides the possible influence of cultural beliefs and unfavourable perception of hospital care, it was also unclear why maternal education had a significant impact on knowledge and accurate recognition of NNJ but not on recourse to self-treatment or planned future hospital visit for a jaundiced infant. This is against the backdrop of other reports from one centre in Southwest Nigeria that associated high maternal education with appropriate health-seeking behaviour for NNJ [21]. Additionally, while another study from Southeast Nigeria found statistically significant difference between mothers with primary education and those with tertiary education with regards to correct perception of NNJ treatment, no significant difference was found between those with secondary and tertiary education [27]. While further research would still be useful to understand fully why women with prior experience of a jaundiced infant will resort to self-treatment and will not seek future hospital care for the same condition, maternal education by health workers must necessarily be complemented with improvement in the quality of hospital care. Some practical and low-cost approaches to making phototherapy more effective which can be readily implemented in resource-constrained settings have been documented [5,35].

A number of limitations of this hospital-based study among urban women are worth noting. For instance, notwithstanding the multi-centre design, it is difficult to ascertain the extent to which the findings in this study can be generalised to the entire country especially among the rural population. We recognise that the exact course of future action that participants of this study would take when confronted with a life case of an infant with jaundice may differ from the information provided in this report. There is also the likelihood of selection bias for the three centres and locations used for this study. Nevertheless, the key findings in this study are consistent with prior reports from Nigeria and transcend cultural and religious affiliations.

## Conclusion

There is a high awareness of NNJ generally among Nigerian mothers and a good proportion especially those who are multiparous or with tertiary education are likely to recognise the onset of the condition in their infants accurately and seek hospital care owing mainly to the education received from professional health workers. Notwithstanding the awareness of NNJ, some mothers, including those with prior experience with a jaundiced infant, are still likely to resort to self-treatment with potentially harmful therapies. While concerted efforts to improve care-seeking practices among mothers are warranted, it is (ethically) imperative for hospital administrators and service providers to ensure that all requisite facilities are optimally functional to protect every infant from the fatal or life-long complications of severe NNJ if and when mothers make the right decision to seek hospital care timely for their jaundiced infants.

## Competing interests

The authors declare that they have no competing interests.

## Authors’ contributions

CVE and NKE conceived and designed the pilot study that formed the basis for the survey instrument used in this study. The final instrument was designed by BOO and reviewed by CVE, MMY and ROU who also personally supervised data collection from the participating centres. BOO analysed the data and drafted the manuscript. All other authors critically reviewed the draft, read and approved the final manuscript.

## Pre-publication history

The pre-publication history for this paper can be accessed here:

http://www.biomedcentral.com/1472-6963/14/192/prepub

## Supplementary Material

Additional file 1 Multi-centre maternal survey on neonatal jaundice. Click here for file
