# Supplementary material for: Pattern and predictors of maternal care-seeking practices for severe neonatal jaundice in Nigeria: a multi-centre survey
Source: BMC Health Serv Res. 2014 Apr 28;14:192. doi: 10.1186/1472-6963-14-192 (PMC4032169; doi:10.1186/1472-6963-14-192)
Supplement: Additional file 1 — Multi-centre maternal survey on neonatal jaundice. [file 1472-6963-14-192-S1.pdf]

MULTI-CENTRE MATERNAL SURVEY ON NEONATAL JAUNDICE

Instruction: All parts (A, B, C & D) of this form must be duly completed by the respondent/interviewer **ID. NO…………**

HOSPITAL: …………………………………………………………… TOWN ……………………… STATE………………

PART A: RESPONDENT'S PROFILE (Tick or describe as applicable)

1. Age: …. or Group: <20☐ 20-35☐ >35☐ Yrs **2. Marital Status:** Single ☐ Married ☐Separated/Divorced ☐ Widow ☐

3. Ethnicity: Yoruba ☐ Hausa ☐ Ibo ☐ Other………………… **4. Religion:** Christian ☐ Muslim ☐ Other ☐

5. No. of Children: ……….. **6. No. Alive:** ……… **7. Any Twins:** Yes ☐ No ☐ **8. Age of Last Child:** …… (Years)

9. Housing Type: Shared ☐ Self-contained ☐ **10. House Ownership:** Rented ☐ Owned ☐ Inherited ☐

11. Your Education: None ☐ Primary ☐ Secondary ☐ Technical/Teachers College ☐ University ☐

12. Husband's Education: None ☐ Primary ☐ Secondary ☐ Technical/Teachers College ☐ University ☐

13. Your Occupation: ……………………………………… **14. Husband's Occupation:** ………………………………..

PART B: AWARENESS OF NEONATAL JAUNDICE (Tick or describe as applicable)

15. Have you heard about jaundice in babies? Yes ☐ No ☐

16. If Yes for 15, from what source: Doctor ☐ Nurse/Midwife ☐ Friend/Neighbour ☐ Relation ☐ Radio/TV ☐ Other ☐

17. Can you recognise a baby with jaundice? Yes ☐ No ☐

18. If Yes for 17, (a) Where in the baby would you look? ……………… (b) What would you see? ……………………………

19. What do you think are the possible effects of jaundice in a baby?

Poor/Not feeding ☐ Irritable ☐ Abnormal cry ☐ Abnormal body stretching ☐ Abnormal eye movement ☐ Other……….

20**. What are the possible dangers of jaundice?**

Death ☐ Motor deficit/disability ☐ Delayed milestones ☐ Mental retardation ☐ Speech defects ☐ Deafness ☐ Other ☐

21. What do you think are the possible causes of jaundice? ……………………………………………………………...

22. Where do you plan to have your baby? This hospital ☐ **Other**: Private hospital ☐ Govt hospital ☐ TBA ☐ Home ☐

23. Which of these items are you likely to use? Dusting Powder ☐ Robb ☐ Eucalyptus Oil ☐ Camphor (for clothes) ☐

PART C: EXPERIENCE WITH A JAUNDICED BABY (Tick or describe as applicable)

24. Have you had a baby with jaundice? a) Yes ☐ No ☐ b) **One of Twins?** Yes ☐ No ☐

25. If Yes for 24, who first discovered the jaundice? Self ☐ Relation/Neighbour ☐ Doctor/Nurse ☐ Other ………………

26. What did you do for the baby? ....

27. What treatment was given to the baby? a)………………………………………………b) **How long?** ....(Days)

28. What was the final outcome? Baby died ☐ Baby recovered fully ☐ Baby recovered with complications* ☐

*Nature of complications: ……………………………………………………………………………………

29. If you suspect or detect jaundice in your baby or any other baby what would you do or advise?

……

PART D: FOR INTERVIEWER ONLY. Use back of this sheet for any additional respondent's information

Respondent's consent obtained: Yes ☐ No ☐ **Reason for declining**: ……………………………………………

Interviewer's Name: ……………………………………………… Status ………………… Date ………………………

© NEONATAL JAUNDICE STUDY GROUP OF NIGERIA
